# Multimodality Imaging Diagnosis in Infective Endocarditis

**DOI:** 10.3390/life14010054

**Published:** 2023-12-28

**Authors:** Elena Emilia Babes, Cristiana Bustea, Tiberia Ioana Ilias, Victor Vlad Babes, Silvia-Ana Luca, Constantin Tudor Luca, Andrei-Flavius Radu, Alexandra Georgiana Tarce, Alexa Florina Bungau, Cristian Bustea

**Affiliations:** 1 Doctoral School of Biomedical Sciences, University of Oradea, 410087 Oradea, Romania; eebabes@uoradea.ro (E.E.B.); andreiflavius.radu@gmail.com (A.-F.R.); prada.alexaflorina@student.uoradea.ro (A.F.B.); 2 Department of Medical Disciplines, Faculty of Medicine and Pharmacy, University of Oradea, 410073 Oradea, Romania; ioana.ilias@didactic.uoradea.ro (T.I.I.); babesvlad@gmail.com (V.V.B.); 3 Department of Preclinical Disciplines, Faculty of Medicine and Pharmacy, University of Oradea, 410073 Oradea, Romania; 4 Department of Cardiology, “Victor Babes” University of Medicine and Pharmacy, 300041 Timisoara, Romania; silvia.luca0@yahoo.com; 5 Institute of Cardiovascular Diseases Timisoara, 300310 Timisoara, Romania; 6 Research Center of the Institute of Cardiovascular Diseases Timisoara, 300310 Timisoara, Romania; 7 Medicine Program of Study, Faculty of Medicine and Pharmacy, University of Oradea, 410073 Oradea, Romania; tarce.alexandrageorgiana@student.uoradea.ro; 8 Department of Surgery, Oradea County Emergency Clinical Hospital, 410169 Oradea, Romania; cristibustea@yahoo.com

**Keywords:** infective endocarditis, prosthetic valve endocarditis, native valve endocarditis, cardiac implantable electronic device infection, radiolabelled WBC, 18-fluorodeoxyglucose PET/CT, echocardiography, cardiac CT

## Abstract

Imaging is an important tool in the diagnosis and management of infective endocarditis (IE). Echocardiography is an essential examination, especially in native valve endocarditis (NVE), but its diagnostic accuracy is reduced in prosthetic valve endocarditis (PVE). The diagnostic ability is superior for transoesophageal echocardiography (TEE), but a negative test cannot exclude PVE. Both transthoracic echocardiography (TTE) and TEE can provide normal or inconclusive findings in up to 30% of cases, especially in patients with prosthetic devices. New advanced non-invasive imaging tests are increasingly used in the diagnosis of IE. Nuclear medicine imaging techniques have demonstrated their superiority over TEE for the diagnosis of PVE and cardiac implantable electronic device infective endocarditis (CIED-IE). Cardiac computed tomography angiography imaging is useful in PVE cases with inconclusive TTE and TEE investigations and for the evaluation of paravalvular complications. In the present review, imaging tools are described with their values and limitations for improving diagnosis in NVE, PVE and CIED-IE. Current knowledge about multimodality imaging approaches in IE and imaging methods to assess the local and distant complications of IE is also reviewed. Furthermore, a potential diagnostic work-up for different clinical scenarios is described. However, further studies are essential for refining diagnostic and management approaches in infective endocarditis, addressing limitations and optimizing advanced imaging techniques across different clinical scenarios.

## 1. Introduction

Cardiovascular disorders exert a major impact on public health and the worldwide economy owing to their considerable expenses. Published scientific investigations have definitively demonstrated a causal association between cardiovascular risk factors and both particular clinical and preclinical conditions, including heart failure, stroke, arterial stiffness, infective endocarditis, etc. [1].

Infective endocarditis (IE) is an infection of the endocardium that may affect native heart valves, implanted prosthetic valves or various cardiac devices [2]. The incidence of IE is approximately 15 cases/100,000 population with a progressive increase registered over the last years. Despite all the advances in the diagnosis and management of the disease, mortality remains high, with up to 14–22% in-hospital mortality and up to 40% 1-year mortality [3,4]. There has been an increase in the incidence of prosthetic valve endocarditis (PVE) over the last few years, accounting for 20–30% of all cases of IE [5,6,7]. Also, IE induces increased myocardial production of hydrogen peroxide H_2_O_2_ and the formation of thiobarbituric acid reactive substances [8], providing evidence for the presence of oxidative stress in the heart [9].

The risk of developing IE remains high among patients with a previous history of IE, patients with surgically or transcatheter implanted prosthetic valves or with prosthetic material used for valve repairs, patients with untreated or incomplete repair of cyanotic congenital heart disease, those with surgically implanted prosthetic material (valved conduits) and patients with left ventricular assist devices [6].

Early and accurate diagnosis is critical in IE and will have an important impact on the outcome. A delayed or missed diagnosis can have catastrophic consequences: heart failure, abscess formation, atrioventricular conduction abnormalities, prosthetic valve dysfunction and embolic events. The modified Duke criteria are in use and can classify patients into one of three categories: definite, possible or rejected. Imaging plays an important role in patients with IE, and elements described with different imagistic techniques are part of the diagnostic criteria [10,11,12]. Current data support the role of the multidisciplinary approach in IE by a specialised endocarditis team that should include cardiologists, cardiac surgeons, infectious disease specialists, microbiologists and imaging specialists for improved management and outcome in IE [6,13]. Cardiovascular imaging has become very complex with an increasing role in the diagnosis of IE. Cardiologists trained in multimodality imaging, but also radiology and nuclear medicine specialists, are currently key members in the Endocarditis Team [6].

Echocardiography remains the first-line test, but it can be normal or inconclusive in up to one-third of cases, especially in PVE or cardiac implantable electronic device infective endocarditis (CIED-IE). Transthoracic echocardiography (TTE) and transesophageal echocardiography (TEE) are essential techniques and can depict major imagistic criteria for diagnosis such as vegetations, abscesses, pseudoaneurysms, intracardiac fistulas, valvular perforations or aneurysms and new dehiscence of a prosthetic valve [12,14].

The modified Duke criteria have a sensitivity and specificity of approximately 80% for native-valve IE (NVE) and significantly less for prosthetic material IE. New imaging techniques are required to improve diagnosis and consequently treatment and outcome [7,15,16,17,18,19,20]. Imaging tools like cardiac computed tomography angiography (CTA), 18-fluorodeoxyglucose positron emission tomography/computed tomography (18 F-FDG PET-CT) and radiolabelled white-blood-cell single-photon emission tomography combined with computed tomography (WBC SPECT/CT) can reveal major criteria for diagnosis [21].

These new tests will give complementary information to echocardiography and can improve diagnostic accuracy but are also able to evaluate the severity and the extent of the infection and perform a preoperative evaluation. In the absence of a definite diagnosis after TTE and TEE, multidetector CTA and nuclear imaging techniques such as 18 F FDG PET/CT or WBC SPECT/CT can reduce the rate of misdiagnosed IE. These new imagistic tools are particularly required in the setting of PVE, the paravalvular extension of infection and cardiac implantable electronic device infective endocarditis (CIED-IE). ECG gated CTA can visualise in 3D or 4D heart valves and perivalvular tissue and can accurately identify the perivalvular extension of infection, respectively, abscesses and pseudoaneurysms [22]. The evaluation of the aortic valve and root and detection of coronary artery embolic complications can be achieved with cardiac CTA, providing important information for surgical planning. In cases with prosthetic valves with or without aortic duct prosthesis, adding CTA is advised [23].

In patients with prosthetic valves, pacemakers, internal cardioverter defibrillators (ICDs) and left ventricular assist devices (LVADs), 18 F-FDG-PET/CT has demonstrated an additional diagnostic value for cardiac infection detection but also for the detection of extracardiac infectious foci in NVE and PVE [16,24]. WBC SPECT/CT is an investigation with increased specificity but with low sensitivity and many disadvantages correlated with patient preparation and comfort. The investigation is a potential approach in patients with suspected PVE with inconclusive echocardiography. In these patients, 18 F-FDG-PET/CT is recommended as first-line investigation due to its high sensitivity in detecting active infection. In situations with inconclusive results for 18 F-FDG-PET/CT, WBC SPECT/CT is recommended due to its high specificity. In CIED-IE, 18 F-FDG-PET/CT and WBC SPECT/CT can add to the diagnosis. Pocket infections can be detected with high sensitivity by FDG-PET/CT, but for lead infections, the sensitivity is reduced [25]. Multimodality imaging has an increasing role in the diagnosis of IE. A correct imaging evaluation is dependent on the informed use of the imaging tools.

The present paper aims to assess the role of multimodal imaging in diagnosing infective endocarditis, specifically native valve endocarditis (NVE), prosthetic valve endocarditis (PVE) and CIED-IE. Our manuscript contributes insights into the strengths and limitations of various imaging tools, emphasizing their informed use in clinical practice. It provides a current understanding of a multimodal imaging approach, offering valuable diagnostic strategies for different clinical scenarios. Novel aspects are evaluated, including the diagnostic value of advanced imaging techniques (e.g., cardiac CTA, FDG PET-CT and WBC SPECT/CT), particularly in challenging medical contexts like PVE and CIED-IE with inconclusive echocardiography.

## 2. Methodological Approaches

Extensive investigation of the literature was accomplished by researching scientific information regarding imaging diagnostics in IE on native valves, prosthetic valves and cardiac devices and the multimodality imaging diagnostic approach. Keywords, MeSH terms and Boolean operators were used for literature selection by investigating scientific databases: PubMed, Cochrane Library and Web of Science. Non-English, non-informative and non-article or -book publications were excluded during the initial screening process. A set of 153 bibliographic references spanning the years 1986 to 2023 was carefully selected and cited to validate the scientific data presented in this paper (the latest literature search was made on 15 November 2023).

**Figure 1:**
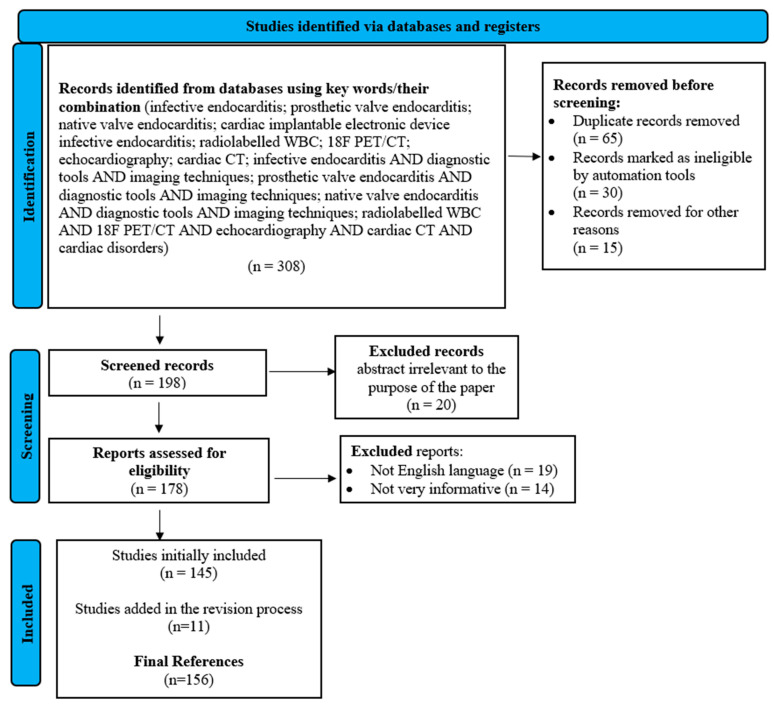
PRISMA flowchart describing literature selection.

The following PRISMA flowchart (Figure 1) represents the methodology for identifying the studies included in this paper, designed based on recommendations provided by Page et al. [26].

## 3. Transthoracic and Transesophageal Echocardiography

Echocardiography is the imaging technique of choice and is the first performed as soon as IE is suspected [27,28,29]. Echocardiographic characteristic findings that represent major criteria for diagnosis are vegetation, abscess, pseudoaneurysm, intracardiac fistula, valvular perforation or aneurysm and/or new dehiscence of a prosthetic valve. Vegetations appear as intracardiac masses attached to valves or intracardiac devices, oscillating or not. Abscesses are non-homogenous irregular perivalvular masses, while pseudoaneurysms are perivalvular pulsatile areas that communicate with cardiac chambers. Leaflet perforation is defects in leaflet tissue with colour flow across the defect, while aneurysm is seen as an outpouching of the leaflet. Fistula is a communication between two cardiac chambers. Prosthetic valve dehiscence is seen as a paravalvular regurgitation with possible abnormal rocking motion of the prosthetic valve [30].

TTE is the initial method of investigation followed by TEE for further characterisation of lesions or identification of complications (except right-sided IE with a good-quality, clear transthoracic image) [6]. In most cases, IE cannot be excluded with TTE. With the exception of patients without prosthetic valves with a conclusively negative optimal image, all other patients will require further evaluation with TEE [31].

In the case of prosthetic heart valves or intracardiac devices, or if TTE is non-diagnostic but the suspicion of IE persists, TEE is necessary [28]. For IE detection in patients with different types of bacteraemia, risk scores have been developed to identify those cases that should be evaluated by echocardiography [6]. A repeat echocardiography may be necessary if the initial examination is negative but a high suspicion of IE is ongoing, with an optimal timing recommended at 5–7 days by ESC guidelines and 3–5 days by AHA guidelines [6,7,32], and in patients with a positive diagnosis of IE at a high risk of complications (PVE, aggressive microorganisms) [6]. New complications such as an embolism, heart failure, new murmur, atrioventricular block, abscess and persisting fever are other indications for repeating echocardiography [33]. In forms of uncomplicated IE, we should consider repeating echocardiography to observe subclinical complications and the evolution of vegetation size. TEE is recommended before switching from parenteral to oral therapy, and TTE or TEE is needed to assess valve morphological and functional status at the completion of antibiotic therapy [6].

TTE can evaluate native left-sided-valve IE, tricuspid-valve IE and anterior aortic abscess. TTE has a sensitivity of 65% in detecting vegetations, but it is less accurate in detecting paravalvular complications such as perforations, abscesses and fistulae [12,34,35].

TEE is superior in identifying and quantifying vegetations, as the size of vegetations will determine the risk of embolic events and the indication for early surgery. TEE is the gold-standard imaging in IE having a sensitivity between 90 and 100% and specificity of 90% for NVE and lower for PVE and CIED-IE. The echocardiographic differential diagnosis between vegetations and other intracardiac masses or ultrasound artefacts remains a challenge. Various pathologies such as myxomatous mitral valve, papillary fibroelastoma, thrombus, nonbacterial thrombotic endocarditis and Lambl excrescences can be confounded with vegetations. Furthermore, a normal echocardiography does not exclude IE. Degenerative valvular changes, the presence of prosthetic material or a cardiac device may impair the visualisation of lesions due to IE [36,37].

For the right-heart IE, TTE is the first line diagnosis, and TEE is required in a minority of cases (when TTE is equivocal or there is a prosthetic valve or an intracardiac device). Because the tricuspid valve is located anteriorly and usually the vegetations are larger in the right heart, they can be detected equally well with TTE as with TEE [38,39]. The right heart has a particular anatomy, and sometimes the differential diagnosis of vegetations may be difficult [39]. Vegetations located on the pulmonary valves and coexistent left-heart IE are better detected with TEE, but in general, right-ventricle-outflow-tract and pulmonic-valve IE is difficult to image with TTE and even with TEE. TEE is also more sensitive in the evaluation of intravenous catheters, devices, prosthetic valves and IE complications (perivalvular abscesses) [38]. Tricuspid-valve IE can be imaged with TTE, but in general, several views are required to evaluate all three leaflets and the tricuspid ring. Three-dimensional TEE can visualise better the tricuspid valve apparatus and surrounding tissue versus two-dimensional TEE, which can more accurately identify and quantify vegetations on the tricuspid valve. In patients with a tricuspid ring, valve prosthesis or intracardiac devices, 3D echocardiography is superior in detecting vegetations and their location and in guiding therapeutical interventions of device extraction [40].

The evaluation of PVE is difficult due to artefacts from acoustic shadowing, making the visualisation of vegetations and paravalvular extension more difficult. Furthermore, postoperative anatomy is changed especially in the first period when oedema or hematoma may be present. PVE can affect the frame and strut leaflets and may involve the perivalvular space. Echocardiography and blood cultures, the basic elements for diagnosis, are frequently negative in PVE. TTE has a reduced sensitivity of 36–69% for the detection of vegetations in PVE, and for paravalvular complications, the sensitivity and specificity are lower. TEE is superior, having a sensitivity of 86–94% and a specificity of 88–100%, in detecting vegetations. In suspected PVE, echocardiographic detection of valve dehiscence and paravalvular regurgitation around the prosthetic valve, valve instability and the paravalvular extension of infection require off-axis imaging planes, with multiplanar imaging and 3D echocardiography, when available. For aortic PVE, anterior abscesses are difficult to detect on TEE due to acoustic shadowing, and posterior abscesses are difficult to evaluate with TTE. Complementary use of both techniques is recommended. Endocarditis in atypical locations, for example, in the atrial septal closure, aortotomy or suture site, will require nonstandard echocardiographic evaluation [6].

Perivalvular complications are more frequent in PVE compared to NVE and in aortic-valve IE versus mitral-valve IE. The extension of the infection is more common in the mitral-aortic intervalvular fibrosa for aortic-valve IE, but mitral-valve IE extends more frequently posteriorly and laterally. Clinical signs that raise the suspicion of perivalvular extension are a persistent fever, conduction abnormalities and a new murmur. TEE is superior to TTE for the evaluation of perivalvular complications. TEE is mandatory in PVE, but the diagnostic accuracy is lower than for NVE. Therefore, further multimodality imaging (CTA and nuclear techniques) is required when the suspicion is high and TTE/TEE is negative or inconclusive. The detection of paravalvular extension, which is present in 50% of PVE, can be discovered with these new imaging tests. Paravalvular regurgitation, persistent fever or conduction abnormalities are all indications for CTA or nuclear imaging tests [6,32].

CIED-IE is associated with high mortality, and removal is recommended in all proven cases. The diagnosis based on modified Duke criteria is suboptimal. CIED infection refers primarily to pacemakers and implantable defibrillators but also to left atrial appendage occluders, septal defect closure devices and devices used in nonvalvular heart intervention. These devices represent challenges due to acoustic shadowing and due to the fact that standard echo views are not optimised to image them, so nonstandard views are recommended. Right-heart devices might have an adherent thrombus due to low pressure which is difficult to differentiate from vegetations. TEE is more accurate than TTE for the evaluation of intra- and extracardiac leads, being superior also in depicting perforations, abscesses and fistulae [36,41].

The sensitivity of TEE is significantly higher versus TTE in detecting CIED infections (90 vs. 22–43%). Device-related infection on the leads from the right atrium or right ventricle may be seen on TTE, but TEE should be performed for better evaluation of the right atrium and superior vena cava portions of the leads. For CIED, TTE has poor sensitivity and specificity versus TEE and 3D echocardiography. TTE and TEE provide complementary information in CIED infections. TTE can better identify prognostic features such as ventricular failure, pericardial effusion and increased pulmonary arterial pressure. TEE is better in detecting and quantifying vegetations [42].

The diagnosis of CIED infection is commonly difficult despite the use of both TTE and TEE, especially for the differentiation from a thrombus [4,7,37,43].

Up to 30% of cases with IE can be missed with TTE and TEE, especially in patients with pre-existent severe valvular disease, PV and CIED, small vegetations, abscesses and vegetations that are already embolised. A negative test cannot rule out an infection of the extracardiac part of a CIED [4].

The limitations of TEE are the difficulty in differentiating between an active infection and postoperative changes in patients recently operated and of vegetations from thrombus or fibrous strands [44]. In patients with CIED, artefacts due to acoustic shadowing make it difficult to evaluate the right heart, and pacemaker leads can be infected despite the fact that we cannot detect any vegetation [36].

Three-dimensional echocardiography (3D) and intracardiac echography have an increased role in these situations [29,45]. Three-dimensional echocardiography is performed with a multiplanar probe that contains a three-dimensional matrix array. The technique can evaluate vegetations and valves in planes and at angles that are not available with 2D TEE [46]. Three-dimensional echocardiography is especially good for assessing paravalvular abscess, regurgitation and perforation of valves and prosthetic valve dehiscence, being more specific for the exclusion of IE (up to 100%), although not more sensitive than TEE [47,48,49]. Three-dimensional TEE can accurately measure vegetation size [50] and permit more correct surgery planning, providing also important information in the intraoperative phase. Intraoperative TEE is widely used in operating rooms and can affect surgical management in up to 30% of cases [51,52]. Studies to evaluate 3D TEE are limited. The technique should be used as an additional investigation to TTE/2D TEE, as due to a low frame rate, it can miss small highly mobile vegetations [14].

Intracardiac echocardiography involves a catheter equipped with a transducer, inserted in the femoral vein to visualise the intracardiac structures. Intracardiac echocardiography seems to be very sensitive in detecting vegetations on cardiac devices [38]. A prospective study on patients referred for lead extraction for device infection found that intracardiac echocardiography performs better than TEE in identifying intracardiac masses, having a high diagnostic accuracy (sensitivity 100%, specificity 82.8%, positive predictive value 65.6% and negative predictive value 100%, *p* < 0.001). The specificity of intracardiac echocardiography for IE may be reduced because of false-positive results determined by thrombi, strands and noninfective vegetations. The advantages of intracardiac echocardiography need to be confirmed in further studies [53].

In conclusion, TTE is the first-line imaging test used to identify vegetations and the associated valve lesions (Class of recommendations I, Level of evidence B). Due to the limited sensitivity of TTE, TEE has a strong recommendation in the case of inconclusive or negative TTE (Class of recommendations I, Level of evidence B) [6]. Furthermore, TEE has increased accuracy in the evaluation of vegetations and complications of IE. In PVE, both TTE and TEE are recommended, but false-negative results are more common. TEE can be a first-step investigation in PVE, but it is also indicated in the case of negative TTE in PVE and for the detection of periprosthetic abscesses and leaks. In CIED-related infections, TTE and TEE have an important role in the initial vegetation evaluation in infections involving the intracardiac and superior vena cava initial segments of the leads but a minor role for pocket-related infections. A negative TTE and TEE cannot exclude the presence of infection in cases of CIED [54].

## 4. Multidetector Cardiac Computed Tomographic Angiography

Cardiac CTA has class IB recommendation in ESC guidelines for valvular lesion detection and positive diagnosis and for the detection of paravalvular and periprosthetic complications if echocardiography is not conclusive. The accuracy of cardiac CTA is superior to TEE in the evaluation of perivalvular and periprosthetic complications (abscesses and pseudoaneurysms). TEE remains superior for the detection of vegetations, leaflet perforation and fistulae [6].

A large number of studies were performed to assess the accuracy of cardiac CTA in vegetation detection. In cardiac CTA, vegetations are seen as low-to-intermediate attenuation structures or as focal thickening of the valve leaflets [55]. A study that compared multidetector cardiac CTA with TEE and intraoperative findings revealed that CTA can identify 97% of patients who had valve abnormalities in TEE and correctly identify 96% of patients who had vegetations confirmed intraoperatively. Furthermore, multidetector cardiac CTA was able to identify a vegetation attached to a mechanical valve missed by TEE and could differentiate valve calcifications from vegetations [22].

In another study on 49 patients, 12 of them with PVE, 4D cardiac CTA detected vegetations with a sensitivity of 91% [56]. A lower sensitivity of 71% and a specificity of 100% were described for cardiac CTA in depicting aortic valve vegetations (100% sensitivity for vegetations larger than 10 mm) by comparing 4D cardiac CTA with intraoperative findings for aortic-valve IE in 19 patients [23].

A retrospective review of 137 patients who underwent cardiac CTA before surgery revealed a similar sensitivity of 70% in detecting vegetations [57]. Another retrospective study on 75 patients who underwent cardiac CTA and TEE revealed a higher detection rate of vegetations by TEE (97 vs. 72%); furthermore, small vegetations < 10 mm were frequently underdiagnosed by cardiac CTA (53% vs. 94%) [58].

A systematic review of eight studies that compared TEE with cardiac CTA reported the same higher sensitivity for TEE compared to cardiac CTA in vegetation detection (94% vs. 64%, *p* < 0.001) [59]. Furthermore, in a meta-analysis of 20 studies that included 496 patients, a pooled sensitivity for vegetation detection of 82% for TEE, 88% with TEE and multidetector cardiac CTA and 29% for TTE alone was detected. The pooled sensitivity in detecting periannular complications (abscesses, mycotic aneurysms) was 86% for TEE, 100% for TEE and multidetector cardiac CTA and 36% for TTE alone. Adding ECG gated CTA to TTE or TEE led to an important increase in the sensitivity of vegetation detection (from 63% to 100%) in PVE [60].

The differential diagnosis of vegetations includes thrombi, fibroelastomas and nonbacterial thrombotic endocarditis [61,62]. Fibroelastomas are small hypo-attenuated lesions attached to valves with a thin stalk, usually not associated with valve destruction and incompetence. They are better imaged with TEE due to their small size and high mobility [63]. Nonbacterial thrombotic IE can manifest as small irregular densities in the heart valves, commonly associated with malignancy or autoimmune disease [62].

Perivalvular complications can be detected with increased accuracy by cardiac CTA. Abscesses appear as non-homogenous perivalvular thickening with high echogenicity in echocardiography [6]. In cardiac CTA, a low attenuation central necrotic component with a peripheral enhancing rim is seen [64,65]. A pseudoaneurysm appears as a pulsatile perivalvular anechoic space with evidence of flow and direct communication with the cardiovascular lumen in colour Doppler [55]. In cardiac CTA, a perivalvular contrast filled cavity with a visible connection with the cardiac chambers or the aortic root is described. The contrast agent helps in distinguishing a pseudoaneurysm from an abscess, as the contrast agent will fill the aneurysm cavity [66].

A recent study that evaluated the usefulness of cardiac CTA in detecting perivalvular complications found a sensitivity of 63% for TTE and 90% for TEE for detecting abscesses or pseudoaneurysms, which increased to 100% for both when cardiac CTA was added to the diagnostic [66]. Sims et al., in 137 patients with preoperative cardiac CTA, revealed a sensitivity of 91% for the detection of abscesses or pseudoaneurysms [57]. Four-dimensional cardiac CTA had a sensitivity of 100% and a specificity of 87.5% for pseudoaneurysm detection in patients with aortic-valve IE [23]. A higher sensitivity for cardiac CTA than for TEE for abscess and pseudoaneurysm detection (78% vs. 69%, *p* = 0.052) that increased to 87% for multiphase cardiac CTA studies (*p* = 0.04) was revealed in a systematic review and meta-analysis [59].

Perivalvular extension of IE is more common in PVE and is associated with a poor prognosis. It can lead to the destruction of the valve annulus with valvular dehiscence and perivalvular leaks. TEE is the imaging tool of choice when we evaluate PVE, but cardiac CTA can provide supplementary information when acoustic shadowing caused by the prosthetic material impairs correct visualisation. Prosthetic valve dehiscence can be visualised on cardiac CTA as malalignment between the prosthesis and the annulus and rocking motion on cine images [55,64,65]. Cardiac CTA and TEE have a similar ability to detect valve dehiscence, with a slight superiority of TEE due to colour Doppler that can better visualise paravalvular leaks and due to the ability to better depict rocking of the valve [6]. Compared to TEE, single-phase cardiac CTA has a similar specificity (97 vs. 99%) and a lower sensitivity (46 vs. 15%) for detecting dehiscence [65].

Fistula is an abnormal communication between two neighbouring cavities through an abnormal perforating tract and is usually a consequence of an abscess or pseudoaneurysm. Colour Doppler shows a tract communicating between the two cavities [6]. Cardiac CTA reveals a fistula like a contrast-agent-filled tract interconnecting two neighbouring cavities. TEE is more accurate in detecting fistulas, a complication associated with a poor outcome [67].

Leaflet perforation can lead to severe valvular regurgitation. Leaflet defects can be observed using echo with flow through the defect in colour Doppler. In cardiac CTA, a leaflet defect is seen as a lack of continuity of the valvular leaflet [65,66]. A lower sensitivity was reported for cardiac CTA versus TEE (43% vs. 75%) in detecting leaflet perforation and a higher specificity (89% vs. 79%) [66]. In a study with 29 patients who underwent surgery, all 4 patients with leaflet perforations were missed with cardiac CTA [22]. A higher sensitivity of TEE than cardiac CTA was observed also by Oliviera et al. in detecting valve perforation (81% vs. 41%, *p* = 0.02) [59].

A valve leaflet aneurysm appears as a distorted saccular outpouching and the loss of its homogenous curvature [6,66]. There was a 100% agreement between TEE and CTA for detecting a valve aneurysm [58].

A recent meta-analysis revealed that multidetector cardiac CTA is better in the detection of PVE and paravalvular complications—abscesses and pseudoaneurysms—compared to TEE [68,69]. Studies regarding the role of multidetector cardiac CTA in the diagnosis of PVE revealed a sensitivity of 93%. Added to the standard diagnosis methods, an increased sensitivity of 100% and a specificity of 83% are observed in the diagnosis of PVE, which can modify the strategy of treatment in a quarter of them [60]. It has a certain value as an adjunct to TEE in the evaluation of PVE, being less susceptible to prosthetic valve artefacts [70]. For overall evaluation, ECG gated CTA has a similar diagnostic value as TEE but is superior for perivalvular complications in the diagnostic work-up of PVE [22,23].

Extracardiac findings as embolic events can be detected in CTA, an embolic event being a minor criterion for diagnosis [12]. The peripheral lesions determined by metastatic infection are splenic, renal, hepatic or mesenteric infarctions or abscesses [71], cerebral lesions, mycotic aneurysms, osteoarticular infections and pulmonary septic embolisms determined by the IE of the right heart [72].

In CIED-IE, the sensitivity of cardiac CTA for pacemaker lead vegetation detection is reduced versus TTE or TEE, due to blooming and beam-hardening artefacts [38]. Pacemaker pocket infections can be assessed with a contrast-enhanced CTA scan by describing local peri-device inflammation or abscess collection [71], but cardiac CTA has limited value in pacemaker pocket infections due to difficult differential diagnosis from post recent implantation inflammatory changes [6]. The tricuspid valve is more frequently involved in CIED-IE [73]. In the management of CIED-IE, device extraction and valvular intervention may be necessary [74]. Cardiac CTA can be used for pre-procedural planning to depict the adhesion of leads to neighbouring vasculature. Contrast-enhanced CTA can detect extracardiac septic emboli and mycotic aneurysms, elements that constitute additional criteria in the diagnosis [75].

Cardiac CTA is increasingly used in the diagnosis of IE and its local complications and for the preoperative evaluation of the coronary arteries and the thoracic aorta. ECG gated cardiac CTA with thin section reconstruction is superior to TEE in detecting abscesses and pseudoaneurysms, and a combination of the two methods increases the sensitivity of diagnosis. Cardiac CTA has a good temporal and spatial resolution but is significantly inferior to TEE. TEE is superior to cardiac CTA in detecting small, highly mobile vegetations (<10 mm), leaflet perforations and perivalvular leaks, although cardiac CTA can be a useful adjunct [38]. Furthermore, it can visualise the tricuspid valves and annulus [76]. The tricuspid valve is often difficult to visualise with TEE because it is located anteriorly, and TTE imaging is commonly inadequate because the tricuspid leaflet is thinner and the annulus is saddle-shaped [77]. Furthermore, TTE and TEE have a low sensitivity in abscess detection, especially in patients with prosthetic valves or intracardiac devices [78]. There is also a prognostic role of multidetector cardiac CTA in IE described by Wang et al. in their study and an important role together with TEE in surgery planning and in predicting mortality [79]. Cardiac CTA can increase diagnostic accuracy, especially by detecting perivalvular and periprosthetic complications, and is recommended if TEE is not conclusive or contraindicated both in NVE and PVE. Another advantage of CTA is the detection of distant lesions and portal of entries or the revealing of an alternative diagnosis by whole-body and -brain imaging. But the preferred imaging method for these situations is PET/CT. A mycotic arterial aneurysm located anywhere in the vascular tree including the central nervous system can be detected with CTA. The evaluation of a neurological complication, spondylodiscitis and vertebral osteomyelitis is more accurate with MRI [80].

One of the limitations of the method is encountered in patients with atrial fibrillation due to misalignment artefacts. A wide detector or dual-source cardiac CTA can improve accuracy in these situations [81]. Another issue is the common presence of an associated renal dysfunction that could be worsened with contrast agent administration. Furthermore, cardiac CTA can cause significant radiation exposure, although modern systems may reduce this. Multidetector cardiac CTA may omit small native leaflet perforations and highly mobile vegetations due to its inferior temporal resolution versus TEE [22,82].

## 5. Nuclear Imaging Techniques

### 5.1. 18-Fluorodeoxyglucose Positron Emission Tomography/Computed Tomography

An important supplementary tool to be used in difficult cases of suspected IE is 18-fluorodeoxyglucose positron emission tomography/computed tomography (18 F-FDG-PET/CT). This method provides functional information revealing the extent of IE before structural damage appears [83,84]. In recent ESC guidelines, there is a class I B recommendation in the diagnosis of PVE and may be considered (IIb B) in CIED-related IE [6].

Limited data are available regarding FDG-PET/CT in IE. Most studies were performed on small cohorts of patients that included together NVE, PVE and CIED-IE [85,86,87]. The value of FDG-PET for the detection of infections was also highlighted in a few studies addressed to a specific group of patients with PVE [10,24,88].

The method is superior in identifying infection in different areas within the heart, especially in difficult cases like prosthetic valves where TEE can be challenging. It can diminish the number of missed abscesses at initial echocardiographic evaluation [89]. Different studies have revealed that in PVE, 18 F FDG PET/CT has a sensitivity between 73 and 100% and a specificity between 71 and 100%, with a positive predictive value of 67–100% and a negative predictive value of 50–100% [10,24,25,42,82,90]. Combined with the modified Duke criteria, it leads to an increased sensitivity from 52–70% to 91–97% with the maintenance of specificity [10,24]. The sensitivity of the technique improved over time due to important technical progress and the development of acquisition protocols.

A contemporary meta-analysis of 26 studies on 1358 patients showed that in recent studies, the sensitivity increased for all types of IE. The research included PVE and CIED-IE and revealed a sensitivity of 72–86% and a specificity of 83–84% with FDG-PET [91]. Another recent meta-analysis of 13 studies that included 537 patients found that FDG PET/CT is a useful additive diagnostic test in PVE challenging cases. The sensitivity for PVE was reported at 80.5% and the specificity at 73.1% when compared to the modified Duke criteria [92].

A systematic review and meta-analysis with the objective to assess the value of FDG-PET/CT and radiolabelled WBC scintigraphy, for the diagnosis of CIED infection, revealed for FDG-PET/CT a pooled sensitivity of 87% (95% CI, 82–91%) and a pooled specificity of 94% (95% CI, 88–98%). Both nuclear methods yield high sensitivity, specificity and accuracy for the diagnosis of CIED infection. The scientific evidence is stronger for 18 F-FDG PET-CT and more limited for WBC scintigraphy [93]. In a prospective study by combining CTA with FDG PET CT, the diagnostic accuracy was improved, reaching a sensitivity of 92% and a specificity of 91%, with a positive predictive value of 93% and a negative predictive value of 88% in PVE and CIED-IE [24]. A standardised semiquantitative measure of FDG uptake increased sensitivity to 100% without reducing specificity [88]. FDG PET/CT reclassified 76% of patients with possible NVE and PVE/ascending aortic prosthesis infection according to modified Duke criteria into definite IE in a recent study. Extracardiac infectious foci were revealed in the same study in 28% of patients [87].

FDG PET/CT was also studied for its prognostic significance in a prospective study that included 179 patients with suspected IE. A significant correlation was found in patients with PVE between a positive FDG PET CT and adverse events such as unplanned heart surgery and death. Furthermore, in patients with NVE and PVE, a more intense FDG uptake is correlated with an increased incidence of embolic events [94].

PET/CTA findings are a major criterion in the diagnosis of IE in ESC guidelines [6]. Images registered after recent surgery need to be interpreted by taking into account the postoperative early structural and metabolic changes due to postoperative inflammation and avoiding their labelling as a positive pathological result. Previous guidelines recommend postponing PET/CTA to 3 months after surgery [10] although this 3-month period of safety is not based on much scientific evidence, and several studies have questioned it [4]. European Association of Nuclear Medicine guidelines recommend a period of only 1-month minimum interval after surgery. It seems that postoperative inflammation can be differentiated from active infection. Prostheses often present with a characteristic pattern of homogenous and diffuse mild FDG uptake in the postoperative period. This finding combined with the absence of anatomic lesions constitutes the normality pattern [88,95].

The technique and the materials used during surgery have a role in influencing the accuracy of FDG-PET/CT examination [96,97]. A surgical adhesive known as Bio Glue (Cryolife Inc., Kennesaw, GA, USA), used especially in patients with aortic root grafts with a prosthetic valve [88], and the Medtronic Mosaic bioprosthetic mitral valve were reported to be correlated with false-positive results [98].

PET/CTA can depict metabolic and anatomic findings. Anatomical lesions such as vegetations, fistulas, pseudoaneurysms, and abscesses determined by IE can be depicted by CTA. A visual analysis regarding the location and distribution of the FDG uptake, as well as a quantitative evaluation of the intensity of uptake, can be performed. An absent or a homogenous diffuse uptake of FDG is considered normal. PVE can be excluded if there is no FDG uptake. An increased ratio between FDG uptake at the level and around the prosthesis and the background standardised FDG uptake of >4.4 is suggestive of PVE [25]. In general, a focal or diffuse and heterogeneous uptake is a sign of infection and should be considered a major criterion for PVE [6]. A new index was proposed by Roque et al., the valve uptake index (VUI), that can improve the correct interpretation of patterns of distribution and will increase diagnostic ability in PVE. These characteristics are stable for a minimum of 1 year post-surgery, and there is no objective reason to postpone PET/CTA examination [99]. A negative PET/CTA can rule out infection, and this is an important advantage in PVE suspicion [97].

FDG PET/CT should be performed as early as possible in the diagnosis of IE because, after the initiation of antibiotics, the low inflammatory activity can create confounding results [88,100]. Whether prior antibiotic treatment affects the diagnostic accuracy of nuclear imaging methods remains an area of debate. In a recent retrospective study on 153 patients who underwent 171 FDG PET/CT studies, including 119 studies performed while patients were receiving antibiotic therapy, no significant impact on the diagnostic performance of FDG PET/CT studies was found [101,102]. Another study on 80 patients did not reveal any influence of prolonged antibiotic therapy before the procedure on the imaging results [85]. On the other hand, a few other studies have shown a possible decrease in the sensitivity of FDG PET/CT when investigating suspected CIED infection in patients already treated with antibiotics [103,104].

Prior antibiotic therapy had no significant influence on the diagnostic accuracy of labelled WBC SPECT-CT in 319 studies performed on 271 patients with suspected bacterial infections, in whom the sensitivity was 88.7% in 169 patients on antibiotic therapy and 92.1% in those who were not receiving antibiotics [105]. Other studies observed false-negative results in patients with suspected IE [106,107] and in patients with suspected CIED infection [108,109] who received prior treatment.

FDG PET/CT has increased sensitivity but lower specificity because FDG uptake may be more intense due to inflammation of non-infectious aetiology [4,25]. In situations with false-positive results, WBC SPECT-CT or other imaging tests are preferred [4,6,106]. False-positive results may be recorded in recent thrombi [110] and inadequate patient preparation. False-negative results are produced in the case of small-size vegetations, prior antibiotic treatment and elevated blood glucose levels. FDG uptake has a characteristic pattern and distribution type that should be used as diagnostic criteria. Diagnostic accuracy is also affected by the time of scanning [4].

In a patient with suspected PVE, especially if the echocardiographic evaluation is inconsistent, the diagnostic approach will include local evaluation of the heart infection, and this will be a major diagnosis criterion but also include extracardiac assessment to evaluate the distant lesions which will constitute minor criteria. PET/CTA permits the evaluation of the distant lesions and the source of IE or can establish an alternative diagnosis if PVE is excluded [32,88].

The evaluation of distant emboli and foci of infection, with the exception of brain involvement where there is an increased physiologic FDG uptake, is another advantage of 18 F FDG PET/CT [6,10,16,19,20]. Cardiac physiologic uptake may be suppressed with a diet that includes high fat and low carbohydrate intake and/or a prolonged fast before the examination [111]. A retrospective study that focused on extracardiac findings found that 23.6% of patients had extracardiac lesions, and in many of them, this led to treatment modifications [19]. The detection rate of extracardiac infectious lesions in a meta-analysis of 13 studies was 17% and varied with the type of IE, the etiologic agent and the timing of the procedure [92].

The value of FDG-PET in the diagnosis of NVE is reduced, but it can detect the source of infection and the extracardiac complications of NVE [112,113]. In NVE, the role of FDG PET/CT was mostly evaluated in retrospective studies [87,114] and revealed a reduced sensitivity for diagnosis of 14% with a correct diet and even less (6%) without the diet [20,90]. FDG-PET/CT was studied in 64 patients with NVE and in 109 patients with PVE. FDG-PET/CT performed much better in PVE than in NVE, regarding the sensitivity of diagnosis (83% vs. 16%) and as a predictor of a worse outcome [94]. At present, in NVE, 18 F-FDG PET/CT has a limited role in cardiac infection evaluation because the sensitivity of the method is poor but can be used for the detection of a distant septic embolism, which represents a minor criterion for diagnosis [87].

18 F-FDG PET/CT can be considered as an imaging method in patients with CIED-related infections [115]. PET/CT positive results correlated well with the clinical, microbiological and echocardiography findings of device-related infection. The reported accuracy of FDG PET/CT is variable regarding device-related infections with values of 80–89% sensitivity, 86–100% specificity, 94–100% positive predictive value and 85–88% negative predictive value [4,116,117]. A lower accuracy of diagnosis in CIED-IE was reported in a prospective study with a sensitivity of 31% and 63% specificity [104].

Lead infection was detected with a sensitivity of 24–100%, specificity of 79–100%, positive predictive value of 66–100% and 73–100% negative predictive value in different studies. Pocket infection was diagnosed with a sensitivity of 87–91%, specificity of 93–100%, 97% positive predictive value and 81% negative predictive value [104,116,118]. An increased specificity was also revealed in a recent study on 63 patients with suspected CIED infection. For lead infection, the sensitivity was only 38.5% but with an increased specificity of 98% [119].

In a meta-analysis conducted on 14 studies that included almost 500 patients, the pooled sensitivity was 83% and specificity 89%. There was a better diagnostic performance for pocket infection than for lead infection [120]. Another meta-analysis of 11 studies showed a sensitivity of 87% and a pooled specificity of 94% of FDG PET CT for CIED infection [93]. Moreover, the sensitivity and specificity were very good in pocket infection (93–96% and 97–98%, respectively), better compared to the diagnostic accuracy for lead infection and endocarditis [93,121]. The accuracy of FDG PET/CT in the diagnosis of CIED infection will be further evaluated in ENDOTEP, a large French multicentre study [122].

If there is clinical suspicion of device-related infection, an intense and heterogeneous 18 FDG uptake along the leads is a sign of active infection, and a focal hotspot is the best criterion for lead infection [4]. The diagnostic performance is influenced by the protocol used for scanning and patient preparation and the time interval after the implantation of the device. In the first 2 months after implantation, a mild uptake can be observed, but no uptake is registered after 6 months. Scanning 3 h after 18 FDG injection leads to an increased accuracy of diagnosis compared to the 1-hour protocol, especially for lead-related infections (sensitivity 91% and specificity 100% for the device; 61% sensitivity and 79% specificity for the leads; and 94% sensitivity and 100% specificity for the pocket) [116]. The infection of the pocket and the extracardiac portion of the lead is detected with almost 100% accuracy in various studies (sensitivity, specificity and accuracy for the diagnoses of pocket infection were 93%, 98% and 98%, respectively) [38,93,121].

18 F FDG PET/CT has additive diagnostic value to Duke criteria, especially in CIED, being able to visualise the entire device. 18 F-FDG PET-CT has the advantage of detecting multiple sites of infection (pocket/generator, leads) and septic emboli in the same examination, with all the therapeutical consequences [104,122,123]. PET reclassifies 90% of Duke-possible patients with suspected device infections [124]. CIED IE diagnosis with FDG PET/CT with cautious interpretation of data in the first 6–8 weeks after implantation has good accuracy. WBC SPECT/CT is also useful in CIED IE but is less available. However, diagnosis is commonly confirmed by revealing vegetations on the tricuspid or less frequently on the pulmonary valve with TTE combined with TEE. Intracardiac echocardiography can add to diagnosis. Perivalvular extension is rarely observed in right-heart IE. Pulmonary CT is useful for evaluating septic embolisms, pulmonary infarcts or abscess occurrence [6,80,95].

The disadvantages of 18 F-FDG PET-CT are the limited value in the first 2 months after implantation as FDG uptake can be present in the absence of any infection, the high cost, limited availability, radiation, complex patient preparation and the need for trained personnel. There is an increasing number of procedures like TAVI or left ventricular assist devices (LVADs), and IE related to these devices represents a new challenge [125]. Modified Duke criteria and echocardiography in particular have a decreased sensitivity in TAVI IE. The acoustic shadow produced by the valve stent decreases the sensitivity of echocardiographic examination [126,127,128]. FDG PET/CT improves the accuracy of diagnosis in these situations. In TAVI patients with suspected IE, vegetations may be found in the stent frame or outside the valve, mainly on the mitral valve, or no vegetations are found. Multiple imaging with FDG PET/CT(A) and intracardiac echocardiography can add to the accuracy of diagnosis in patients with negative TEE [129,130].

In a small study on 16 patients with suspected TAVI IE, only half of the 10 cases with definite IE were detected with echocardiography while FDG PET CT was positive in 9 of 10 cases [129]. Cardiac CTA or FDG PET/CT had an important role in patients with suspected TAVI IE in a retrospective multicentre study. The diagnosis was modified in one-third of patients after adding the two diagnostic tools to the modified Duke criteria [130].

If TAVI itself can cause an inflammatory reaction after the implantation procedure and can cause increased FDG uptake was a question to be answered in a small study that compared FDG uptake within 1 month after TAVI in a control group (31 patients) versus 14 patients with suspected TAVI IE. In the control group, seven patients (22%) had FDG uptake. In all seven patients with definite IE and in one case with rejected IE, FDG uptake was registered. A focal pattern of the uptake with less than 25% of the valve circumference affected signified true infection. A diffuse uptake that affected more than 50% of the circumference was observed in the control group and in the rejected case [84]. Further studies should investigate how long an increased uptake persists after TAVI and the prognostic value of FDG PET CT in this situation.

Infection of LVADs is a severe complication associated with a bad prognosis [131]. The site of infection is more commonly at the driveline entry point through the abdominal wall but can progress to deep tissue. Infection of the central components (pump or canula) is difficult to diagnose and is correlated with a worse outcome. Echocardiography has little role due to artefacts, and the role of CCT is limited as well. 18 FDG PET/CT and radiolabelled WBC SPECT/CT are more reliable. The diagnostic performance is higher for FDG PET/CT compared to radiolabelled WBC SPECT/CT (92% vs. 75%) and could be the first-line nuclear medicine procedure [132].

In 28 patients with LVADs, FDG PET/CT was indicated for suspected infection. The magnitude of infection detected by PET CT correlated with prognosis [130]. Another study on 57 patients found similar results with increased mortality when FDG PET/CT revealed extensive involvement of the entire LVAD and the thoracic lymph nodes [133]. If these findings are validated in larger studies, FDG PET CT could be included in the criteria for heart transplantation, with those with widespread infection being prioritised.

FDG PET and leukocyte scintigraphy are more sensitive in detecting IE than echocardiography for CIED (pacemakers, ICDs, resynchronisation therapy devices, LVAD). The method can help in the diagnosis of IE but also provide information about the cardiac lesions, increase the sensitivity in detecting abscesses and help in the decision of surgical treatment [134]. Moreover, 18 F FDG-PET/CT has good spatial resolution, can identify extracardiac complications and has feasible logistic and increased comfort for patients compared to leucocyte scintigraphy which requires laborious preparation and multiple visits of patients and can miss small infectious foci [4].

18 F FDG-PET/CT has high sensitivity in PVE and good accuracy in detecting perivalvular/periprosthetic complications [6]. Multidetector cardiac CTA and 18 F FDG-PET/CT reveal complementary data in patients with IE. While multidetector cardiac CTA reveals mainly anatomical information and can detect with high sensitivity and specificity perivalvular complications and less well vegetations, 18 F-FDG-PET/CT provides functional data and can detect extracardiac involvement. By combining these two imaging tools, an increased diagnostic accuracy is achieved [24]. Added to the standard diagnostic work-up, it can change the management strategy in 25% of cases. When a hybrid PET/CT system is available, 18 F FDG PET CT should be performed together with multidetector cardiac CTA [60].

### 5.2. Radiolabelled Leucocyte SPECT/CT Scintigraphy

Promising results were observed in several research studies regarding the utility of radiolabelled WBC SPECT/CT scintigraphy in cases with high clinical suspicion of PVE without confirmation in microbiological or echocardiographic evaluations. Current ESC guidelines made a class IIa C recommendation in patients with high suspicion of PVE when echocardiography is negative or non-diagnostic and when PET/CTA is not available [6].

While the uptake of 18 Fluorine FDG in PET/CT is related to the rate of intracellular glucose metabolism which is increased in activated inflammatory cells, the increased accumulation of neutrophils at the site of infection is the basis for the diagnostic use of scintigraphy with labelled leukocytes in IE [111,135].

Rouzet et al. compared the two nuclear medicine investigations 18 F FDG PET/CT and WBC SPECT/CT in patients with suspected PVE [25]. The study confirmed the high specificity of labelled WBC SPECT/CT. Moreover, the role of SPECT/CT was especially underlined in the first 2 months after surgery when 18 F FDG PET/CT may produce false-positive results. SPECT/CT permits evaluation of infection as localisation and extension even in the early postoperative period. Furthermore, whole-body imaging allows the diagnosis of distant embolic and metastatic infectious lesions. SPECT/CT studies have high specificity in the diagnosis of PVE, NVE and CIED-IE. Inflammation–infection characterisation with autologous radiolabelled WBC is a highly specialised method that requires highly qualified personnel and multiple and long scintigraphy acquisitions. The sensitivity is limited, affecting its negative predictive value [80].

A stepwise approach is recommended, with FDG-PET/CT used first because it has a high sensitivity, and if the result is not certain, then WBC-SPECT/CT should be added. Both techniques proved similar accuracy in CIED-IE [93,106,108,136,137]. Using an imaging technique with high specificity as leucocyte scintigraphy in a group of patients selected with a high-sensitivity imaging tool is appropriate.

The advantages of both nuclear methods are the ability to evaluate extracardiac areas in a single imaging procedure and reveal extracardiac infection sites as primary infective processes or as a consequence of a septic embolism with the exception of the brain where uptake is intense due to its increased metabolism [4,11,16,138] and to detect the portal of entry [24]. The detection of metastatic infection changes treatment in 35% of patients [20].

## 6. Cardiac Magnetic Resonance Imaging

The role of cardiac magnetic resonance imaging (CMR) in the diagnosis of IE requires further clarification. Theoretically, CMR offers a superior 3D assessment of cardiac structures and morphology compared to echocardiography or CTA. Anatomical and functional data on valvular regurgitation, as well as myocardial involvement with oedema or inflammation of associated myopericarditis, can be revealed with CMR [4,139]. CMR can depict myocardial involvement in IE and can identify vegetations and also the paravalvular extension of infection with delayed contrast enhancement [139,140,141].

A limited number of research studies that studied the role of CMR in IE are available, mostly case series on a reduced number of patients. Dursun et al. aimed to study the utility of CMR for the diagnosis of IE and found that CMR can detect vegetations in patients with suspected IE and can provide valuable diagnostic and prognostic information. Perivalvular involvement was revealed with delayed contrast enhancement, but only 68% of vegetations were depicted [139]. Zatorska et al. studied 20 patients and observed that due to a lower spatial resolution of CMR, vegetation visualisation was limited, but they observed important advantages in detecting the perivalvular extension of infection and in evaluating valvular regurgitation and myocardial inflammation [142].

On the other hand, CMR has a superior ability of tissue characterisation of cardiac masses and can help in differential diagnosis. A recent study that aimed to evaluate the accuracy of CMR to identify vegetations and complications of IE versus echocardiography revealed that all vegetations observed with echocardiography were also visualised with CMR. By tissue characterisation, in some cases, alternative diagnoses were confirmed (e.g., fibroelastoma, non-bacterial thrombotic endocarditis) [143].

In a recent retrospective study, CMR revealed inconclusive results compared to TEE in diagnosing valvular vegetations and in the clinical management of IE, suggesting that CMR cannot be validated as a confident diagnostic tool [144]. Further prospective studies that will address the value of CMR versus TEE for the diagnosis and management of IE are required. Future developments in the field of this rapidly evolving diagnostic method may improve the current disadvantages of CMR concerning temporal and spatial resolution.

CMR is difficult to use in PVE due to artefacts produced particularly by mechanical prostheses. The information is comparable to CTA, and it can detect paravalvular abscesses, pseudoaneurysms and prosthetic valve dehiscence, but spatial resolution and morphological definition are reduced compared to CTA. It can be recommended when CTA is contraindicated or for hemodynamic evaluation. CMR has a limited role in CIED because most devices are incompatible with MRI and diagnostic utility is diminished due to magnetic susceptibility artefacts. The 2017 ACC/AHA discourage its use for diagnosing IE, being not recommended due to a lack of superiority compared with echocardiography or CTA [134]. Current ESC guidelines recommend MRI for the diagnosis of neurological lesions and as a diagnostic modality of choice for spondylodiscitis and vertebral osteomyelitis [6].

Cerebral MRI is the most sensitive method to detect cerebral emboli. It may provide additional diagnostic findings and may change the timing of surgery [145]. AHA guidelines recommend cerebral MRI in patients with neurological symptoms and suspected IE but also in asymptomatic patients with IE prior to valve surgery to evaluate the presence of mycotic aneurysms. In patients with a high suspicion of IE, cerebral MRI can increase the accuracy of Duke criteria by adding a minor criterion [134].

## 7. Advantages and Limitations of Imaging Diagnostic Tests in IE

Diagnostic strategies in suspected IE will include echocardiography as a first-line imaging investigation and also other imaging tests: CT, nuclear imaging and MRI. The major strengths and limitations of different imagistic tools are revealed in Table 1.

## 8. Imaging Diagnostic Approach in Suspected NVE

Echocardiography is the first-line imaging method in IE, providing diagnostic and prognostic data. It has a class I recommendation in AHA/ACC and ESC guidelines, is largely available and without risks and has a low cost [6,134]. TEE is recommended with or without a prior TTE, including when TTE is negative but the suspicion of IE persists. It may be recommended to repeat TTE/TEE in the diagnosis work-up of IE but also in confirmed IE to evaluate complications or the progression of the disease, especially if there is a change in the clinical status [134].

If diagnostic uncertainty persists after TEE, ECG gated cardiac multidetector CT with or without angiography can improve diagnostic accuracy by detecting vegetations, valve perforation or aneurysms and perivalvular involvement (abscesses and pseudoaneurysms), especially in the aortic area [22,66]. Identification of perivalvular lesions on cardiac CT is considered a major criterion for diagnosis. It offers complementary information to echocardiography and is already included in the ESC guidelines with class I recommendation [6].

Echocardiography, primarily TTE, is the initial choice for assessing patients with aortic regurgitation. TTE identifies the regurgitation mechanism and severity and evaluates LV remodeling. In cases of challenging acoustic windows or inconclusive parameters, TEE and CMR serve as valuable adjuncts, providing precise information for surgical decisions. Additionally, CT is useful for thoracic aorta measurement, coronary artery assessment and complications associated with aortic valve endocarditis or prosthesis dysfunction [146].

The pulmonary valve is less frequently imaged among the cardiac valves. Echocardiography remains the primary method for assessing patients with pulmonary regurgitation and pulmonary stenosis. However, information derived from this technique is frequently complemented by CMR and CT [147].

**Figure 2:**
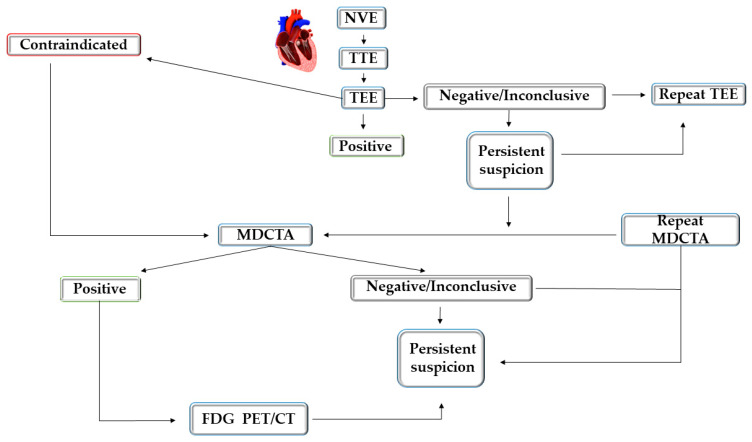
Imagistic diagnostic approach in patients with suspected NVE. NVE, native valve endocarditis; TTE, transthoracic echocardiography; TEE, transesophageal echocardiography; MDCT, multidetector computed tomography; FDG-PET/CT, 18-fluorodeoxyglucose positron emission tomography/computed tomography.

After a negative or inconclusive result with TEE and multidetector CTA but a persistent suspicion of IE, the repetition of TEE and multidetector CTA is recommended. The role of FDG-PET in the diagnosis of NVE is reduced, due to its reduced sensitivity, but it can detect the source of infection and extracardiac complications (distant embolic events) which represent a minor criterion for diagnosis. 18 F FDG PET/CT can be considered in difficult scenarios of NVE, although there is no evidence to support this; it probably has a role when there is a strong clinical suspicion but the Duke criteria are not fulfilled. A negative FDG PET/CT should not be used for the exclusion of NVE. Clearly, echocardiography and multidetector cardiac CTA are the first-choice investigations, but in difficult cases, 18 F FDG PET/CT should be performed within 7 days with correct preparation. In clinical practice, cardiac CT and nuclear imaging tests have a reduced role in NVE but should be considered in the presence of contraindications to TEE. A potential imagistic diagnostic approach in patients with suspected NVE is depicted in Figure 2 [112].

## 9. Imaging Diagnostic Approach in Suspected PVE

PVE presents numerous complications and high mortality. The modified Duke criteria are particularly limited in the case of PVE suspicion as the prosthetic material makes echocardiographic findings hard to interpret. Guidelines recommend TTE as a first-line test for suspected PVE usually combined with TEE, with vegetations being difficult to detect. The image is commonly suboptimal in patients with PVE due to acoustic shadowing. TEE is more sensitive than TTE, but results may be false-negative. If imaging is negative but the suspicion of IE persists, TEE should be repeated 3–7 days later [6,33].

TEE permits better evaluation of PVE, small vegetations and perivalvular abscesses. Three-dimensional TEE can better describe vegetations and perivalvular abscesses, valvular perforations and paravalvular leakage and prosthetic valve dehiscence and provides an improved anatomical localisation of lesions and their relation with the surrounding structures. Multidetector cardiac CTA and nuclear techniques may improve diagnostic accuracy, and this is particularly true in PVE [4].

Multidetector CTA has a high spatial resolution and provides detailed anatomical data [145]. A full 3D dataset scan and post-processing in multiplanar reconstruction permits the evaluation of the prosthetic valve and surrounding structures from any angle [77]. It is particularly useful for the detection of paravalvular complications: abscesses, dehiscence of the prosthetic valve and pseudoaneurysms. Preoperative evaluation and planning can be realised with multidetector cardiac CTA due to detailed anatomical information and the concomitant evaluation of coronary arteries [22,82].

In ESC guidelines, a positive CTA and/or nuclear imaging test (abnormal periprosthetic inflammation detected with 18 F-FDG-PET/CT or by radiolabelled WBC-SPECT/CT) in PVE are major criteria for diagnosis. The confirmation of recent embolic events or an inflammatory aneurysm (clinically silent) is considered a minor criterion. When a hybrid PET/CT system is available, 18 F FDG PET CT should be performed together with multidetector cardiac CTA [6].

**Figure 3:**
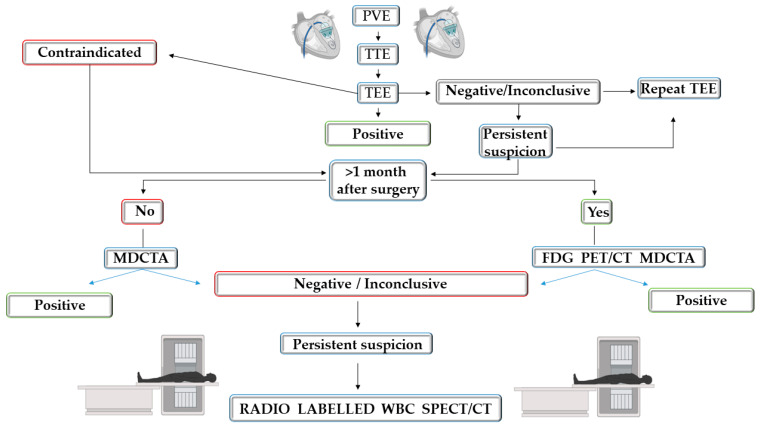
Imagistic diagnostic approach in patients with suspected PVE. PVE, prosthetic valve endocarditis; TTE, transthoracic echocardiography; TEE, transesophageal echocardiography; MDCTA, multidetector computed tomography; FDG-PET/CT, 18-fluorodeoxyglucose positron emission tomography/computed tomography; WBC SPECT/CT, white-blood-cell single-photon emission tomography combined with computed tomography.

When diagnostic uncertainty persists after 18 F-FDG-PET/CT, WBC-SPECT/CT is useful [148]. In patients with suspected PVE, a sequential approach of 18 F FDG-PET/CT followed by WBC SPECT/CT is indicated if echocardiography is not conclusive. Patients with an intense focal cardiac valve uptake at 18 F FDG-PET/CT and those with negative 18 F-FDG-PET/CT need no further investigations. Patients with low diffuse 18 F FDG-PET/CT uptake around the cardiac prosthetic valve will be further investigated with WBC SPECT/CT especially if they are scanned in the first months post cardiac surgery [4]. WBC SPECT/CT can represent an alternative nuclear imaging technique for the diagnosis of PVE when PET/CT is unavailable [6]. A potential imagistic diagnostic approach in patients with suspected PVE is described in Figure 3.

## 10. Imaging Diagnostic Approach in Suspected CIED-Related Infections

A multimodality imaging approach is recommended for the diagnosis of CIED-related infections by the 2020 European Heart Rhythm Association International Consensus and ESC guidelines [6,149]. The imaging methods included are TEE, which can identify CIED-IE, cardiac CTA, which can depict local infection and distant embolic metastatic infections, 18 F FDG PET/CT, which can identify focal uptake and contribute to the positive diagnosis, radiolabelled WBC SPECT/CT, which, similarly to PET, can identify inflammation related to infection, and soft-tissue ultrasound which can depict pathological fluid collections [73].

The diagnosis of pocket infection is often based on clinical examination, by revealing the local signs of inflammation. Soft-tissue ultrasound can help in the evaluation of collections. Pocket infection can coexist with CIED endocarditis. If the diagnosis of pocket infection remains inconclusive, 18F-fluorodeoxyglucose positron emission tomography (18 F-FDG PET/CT) can improve diagnostic accuracy [149]. In cases of positive blood cultures and systemic inflammatory response syndrome when lead infection is suspected, further diagnostic work-up should be performed with TTE and TEE [149,150].

In patients with suspected CIED infection and inconclusive TEE or when TEE is contraindicated, alternative imaging investigations are indicated [71,74]. Nuclear imaging work-up using 18 F-FDG PET-CT is recommended in the 2023 ESC endocarditis guidelines and in the 2020 European Heart Rhythm Association consensus for the management of CIED infections [6,149].

18 F FDG PET/CTA is highly recommended in patients with pocket infection and negative microbiological and echocardiographic results as well as in patients with positive blood cultures and negative echocardiography. The diagnostic accuracy is reduced for lead infection. When tracer uptake is visualised in lead infections, the specificity is very high. However, the sensitivity is low for lead infection, and a negative result does not completely exclude infective endocarditis with small vegetations and low metabolic activity. On the other side, despite being less accurate in diagnosing lead infection or device-related infection, 18 F-FDG-PET/CT has high accuracy for the diagnosis of pocket infection [75].

In 2017, the Heart Rhythm Society guidelines made a weak recommendation for FDG PET CT when the diagnosis of CIED pocket or lead infection is uncertain [74]. The European Heart Rhythm Association (EHRA) guidelines 2020 approved by the European Society of Clinical Microbiology and Infectious Diseases included FDG PET/CT as a major criterion in the diagnosis of CIED-IE. There is a strong recommendation for FDG PET/CT when CIED-IE is suspected, echocardiographic findings are negative, and blood cultures are positive, in all cases with *Staphylococcus aureus* bacteriemia in patients with CIED and for the assessment of embolic localisation and metastatic infection sites [149,151].

Along with CTA, FDG PET/CT is included in the 2017 Appropriate Use Criteria for Multimodality Imaging in Valvular Heart Disease for suspected IE with moderate-high pre-test probability and negative TTE [134]. The current ESC guidelines made PET/CTA a class I recommendation to detect pocket infection and a possible associated pulmonary septic embolism and a class IIb recommendation for lead infection evaluation [6].

**Figure 4:**
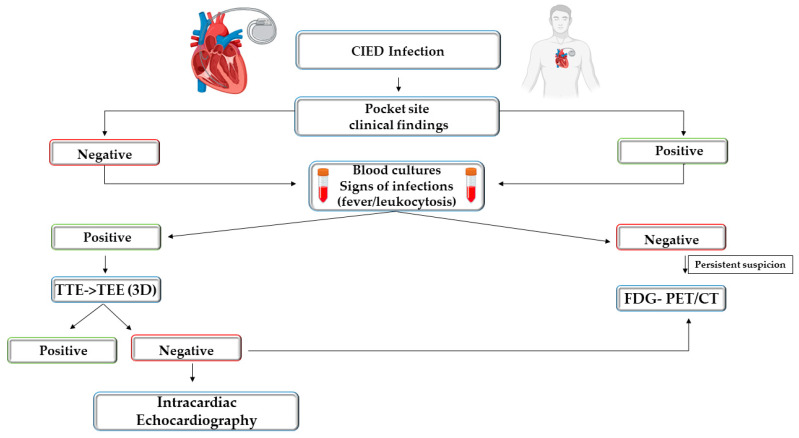
Imagistic diagnostic approach in patients with suspected CIED-related infection. CIED, cardiac implantable electronic device; TTE, transthoracic echocardiography; TEE, transesophageal echocardiography; 3D, three-dimensional; FDG-PET/CT, 18-fluorodeoxyglucose positron emission tomography/computed tomography.

Several research studies are ongoing to discover bacteria-targeting tracers for specific infection imaging, and the research will continue in the future. Until then, 18 F-FDG PET/CT, which depicts the host immune response to infection, should be used for the assessment of CIED-related infections. A potential imagistic approach in CIED-related infection is presented in Figure 4 [152].

## 11. Current Guidelines’ Recommendation for Imaging Investigations in IE

Diagnostic criteria from the current guidelines include findings from new imaging techniques for a more accurate diagnosis. Cardiac native- or prosthetic-valve lesions identified by imaging techniques are major diagnostic criteria of IE. TTE is the first imaging investigation (class I B), followed by TEE in most cases. Indications for TEE are the following: clinical suspicion and negative or inconclusive TTE (class IB); the presence of a prosthetic valve or intracardiac device (class IB); 3–5 days according to the AHA statement [71] or 5–7 days after an initial negative test in the ESC guidelines if the clinical suspicion remains high (class I B); after a confirmed IE by TTE for in-depth characterisation of lesions (except in right-heart IE with a high-quality TTE window (class IC)); for follow-up in different situations that raise the suspicion of complications: murmur, embolic event, abscess, atrioventricular block, persisting bacteriemia or fever or before switching to oral therapy (class IB); at the completion of antibiotic treatment for the evaluation of valvular damage and cardiac function and intraoperative TEE in cases requiring surgery (class IC) [6,32].

CT(A) has a class IB indication in the current ESC guidelines in suspected NVE and PVE for the detection of valvular lesions and positive diagnosis of IE. Furthermore, paravalvular and periprosthetic complications can be detected with cardiac CT(A) in cases with inconclusive echocardiography (class IB) [5]. According to the ACC/AHA guidelines, there is only a moderate strength level of recommendation (class 2a B-NR), with it being reasonable to perform CT imaging if the echocardiographic images are not adequate and we suspect a paravalvular abscess [32].

Nuclear imaging tests, 18 FDG-PET/CT(A), are recommended to detect valvular lesions and confirm a positive diagnosis in PVE (class IB) and may be considered for diagnosis confirmation in patients with CIED-related IE (class IIb B) [6]. In the 2020 ACC/AHA guidelines, it is stated that it is reasonable to perform FDG PET-CT in cases classified as possible IE by modified Duke criteria (2a B-NR) [32]. WBC SPECT/CT should be considered in patients when PVE cannot be confirmed or excluded with echocardiography and PET/CT is not available (class IIa C) [6].

Brain and whole-body imaging (CT, 18 FDG PET/CT and/or MRI) is recommended in symptomatic patients with NVE and PVE to detect peripheral lesions, adding a minor criterion to diagnosis (class IB), and may be considered as a screening test in the absence of symptoms (class IIb B). The following imaging protocol is recommended in the current ESC guidelines for patients with NVE. The initial investigation is TTE/TEE (with repetition in 5–7 days if the first examination is inconclusive and the suspicion of IE persists) (class I). The next imaging test to recommend for a positive diagnosis or for suspected paravalvular complication when TEE is not conclusive is cardiac CTA (class I). Brain and whole-body imaging with MRI, CT, PET/CT and WBC SPECT/CT can be performed to detect distant lesions (class II a). In suspected PVE, the current ESC guidelines recommend as first-step examination TTE and TEE, with repetition in 5–7 days if necessary (class I). The next step is cardiac CT(A) to diagnose valvular lesions or FDG PET/CTA (class I). For suspected paravalvular complications and inconclusive TEE, cardiac CTA should be performed (class I). To detect distant lesions, brain or whole-body imaging (MRI, CT, PET/CT, WBC SPECT) is advised (class IIa). In suspected CIED-IE after initial evaluation with TTE and TEE (with repetition if needed) (class I), further evaluation with PET/CTA to detect pocket infection and/or a pulmonary embolism is recommended (class I). PET/CTA may be considered also for the detection of lead infection (class IIb). A chest CT scan to detect a septic pulmonary embolism has a class II recommendation in the current ESC guidelines and will add a minor criterion in diagnosis [6].

## 12. Advancements and Future Directions in IE Imaging

The development of medical technology will increase the role of multimodal imaging in the assessment of IE. There are multiple technological advancements in imaging that are improving diagnosis, especially in PVE and CIED. As the incidence of PVE and CIED is expected to increase, further studies to investigate multimodality imaging indications and temporal sequencing are needed [153,154].

Echocardiography techniques developed towards a better spatial, temporal and 3D resolution. The use of multidetector CT scanners can improve spatial resolution and reduce radiation exposure. Further improvement in image quality is obtained with the increased use of reconstruction algorithms. An improved ability in the detection of infections can increase the accuracy of diagnosis. The use of new digital detector technology in PET scanners with a long axial field of view greater than or equal to 100 cm will reduce acquisition time and increase the temporal and spatial resolution. Nuclear imaging techniques have in general a long acquisition time, motion artefacts and increased radiation. The accurate evaluation of IE cases is limited by a relatively low spatial resolution of PET and SPECT in conditions of respiratory and cardiac motion. These movements can be modelled using artificial intelligence techniques that can provide image reconstruction by combining previous learned data that compensate for respiratory and cardiac movements. Improved detection of small infectious foci in the area of heart valves corroborated with the findings on echocardiography or CT will improve the accuracy of diagnosis. WBC SPECT imaging has increased specificity, but the quality of the image is inferior due to high levels of noise and low count statistics. Artificial-intelligence-based technologies can be used for image denoising and the improvement of image interpretation [155].

Molecular imaging techniques that use bacteria-specific tracers and antibody tracers against bacterial cell membranes are under development [156].

Image fusion technology can merge complementary information from two or multiple imaging modalities with increased anatomic and functional evaluation of IE findings. CMR has improved regarding spatial and temporal resolution, four-dimensional flow sequences and the reduction in acquisition time. Combined PET/CMR may improve imaging in IE by concomitant anatomical and tissue characterisation with CMR and infection/inflammation detection by PET [14]. Combining PET/CT with a CT angiography can detect metabolic and anatomical findings and is increasingly utilized in patients with complex congenital heart disease and aortic grafts [6].

Future studies should address the existent gaps in imaging diagnosis. One important issue would be establishing the role of [18F] FDG-PET/CT(A) in suspected NVE. The sensitivity of the investigation is low in NVE, and a negative test cannot exclude NVE, but a septic embolism may be identified in specific situations. Furthermore, limited data are available regarding the accuracy of intracardiac echocardiography in the diagnosis of suspected PVE. Another question to be answered is if we should perform a routine imaging screening of a septic embolism, especially of the central nervous system.

## 13. Conclusions

Multimodality imaging is a key element for an accurate and early diagnosis in IE. Every imagistic method has strengths and limitations, but with an appropriate combination of imagistic tools, complementary information is achieved. Echocardiography remains the first-line imagistic investigation, with an increased use of TEE and 3D echocardiography. CTA has an important role in the diagnostic work-up of PVE. CTA is superior to TEE in the evaluation of perivalvular and periprosthetic complications (abscesses and pseudoaneurysms). TEE remains superior for the detection of vegetations, leaflet perforation and fistulae. Nuclear medicine imaging techniques, 18 F-FDG-PET/CT and WBC SPECT/CT, have demonstrated their value for the diagnosis of PVE and CIED-IE and detection of peripheral embolic and metastatic infection sites.

## Figures and Tables

**Table 1:** Advantages and limitations of imagistic methods in IE.

Imaging Method	Advantages	Limitations	Refs.
Cardiac evaluation			
TTE and TEE	Good diagnostic ability in NVE (vegetations, leaks); TEE superior diagnostic ability compared to TTE in PVE and in CIED-IE; Intracardiac and superior vena cava initial segments of the leads can be evaluated in CIED-IE; Evaluation of morphological and functional status of cardiac valves and the hemodynamic consequences; Prognostic value; Embolic risk evaluation; Useful for follow-up; TEE before switching to oral therapy; Widely available; First-line investigation and safe.	Difficult evaluation of perivalvular complications, especially in PVE; Difficult evaluation of right-ventricle outflow tract (pulmonary valve) and anterior structures (tricuspid valve); Not applicable for pocket and extracardiac or extravascular lead infection; Difficult in differentiating lead vegetations from thrombus; Cannot detect peripheral complications; Procedural complications can appear in TEE.	[6,36,44,77,80]
3D echo	Paravalvular abscess; Regurgitation and perforation of the valves; Prosthetic valve dehiscence; 3D TEE can accurately evaluate vegetation size; Surgery planning, intraoperative assessment; Highly specific for exclusion of IE.	Can miss small highly mobile vegetations; Not more sensitive than 2D TEE; Further studies are needed.	[6,14,47,48,49,50]
Intracardiac echocardiography	Very sensitive in detecting vegetations on cardiac devices.	Reduced specificity, false-positive results determined by thrombus, strands; Further studies are needed.	[38,53]
Cardiac CTA	Very good in detection of perivalvular complications (abscess/pseudoaneurysm) (superior to TEE); Acceptable in detection of vegetations, perforations, fistulae (inferior to TEE); CIED-IE patency of venous accesses/soft tissue and infected collections of the pocket; Coronary artery and thoracic aorta preoperative evaluation.	Can miss small vegetations; Cannot evaluate valvular function; Limited diagnostic value for CIED-IE (reduced sensitivity for leads vegetations, lead artefacts); Difficult differentiation between pocket infection and post-implantation inflammatory changes; Atrial fibrillation-misalignment artefacts;Radiation exposure; Risk of nephrotoxicity.	[6,22,38,80,81,82]
[18F] FDG-PET/CT(A)	Increased sensitivity in PVE; Perivalvular/periprosthetic complications in NVE and PVE assessment of the local extension of the infection; IE on other prosthetic materials (after repair surgery in congenital heart disease); CIED-IE good accuracy for pocket and extracardiac or intravascular lead infection; Patency of venous accesses (with CTA).	Low sensitivity in NVE; Can miss small vegetations; Cannot evaluate valvular function; Lower diagnostic accuracy for lead infection; False-negative results in patients with long antibiotic treatment; Limited availability and high cost; Limited value in the first 2 months after implantation; Specific expertise to acquire and analyse images; Radiation exposure; Risk of nephrotoxicity (CTA).	[6,33,35,80]
WBC-SPECT	Increased specificity for PVE, NVE and CIED-IE; Good accuracy for pocket and extracardiac or extravascular lead infection; Permits evaluation of infection in the early postoperative phase.	Limited use for pyogenic infections; Limited sensitivity (low spatial resolution); Long acquisition time; Radiation exposure; Highly trained personnel.	[6,80]
Extracardiac lesion evaluations			
PET/CT and WBC-SPECT whole-body images	Detection of distant embolic lesions (exception of brain); Detection of pulmonary embolic lesions; Detection of the portal of entry; Alternative diagnosis in rejected IE.	-	[4,5,11,16,24,138]
CT(A) and MRI	Detection of distant embolic lesions; Central nervous system embolism, bleeding and aneurysms; Osteoarticular infections or spondylodiscitis; Mycotic aneurysms/pseudoaneurysms.	Radiation exposure and risk of nephrotoxicity (CTA); Restricted use in CIED-IE (most devices incompatible with MRI).	[6,80]

TTE, transthoracic echocardiography; TEE, transoesophageal echocardiography; CT(A), computed tomography angiography; NVE, native valve endocarditis; PVE, prosthetic valve endocarditis; CIED-IE, cardiac implantable electronic device infective endocarditis; [18F] FDG-PET/CT, 18-fluorodeoxyglucose positron emission tomography/computed tomography; MRI, magnetic resonance imaging; WBC SPECT/CT, radiolabelled white-blood-cell single-photon emission tomography combined with computed tomography. Ref, references.

## Data Availability

Data sharing is not applicable.
